# Supplementary material for: The Systems Biology Research Tool: evolvable open-source software
Source: BMC Syst Biol. 2008 Jun 29;2:55. doi: 10.1186/1752-0509-2-55 (PMC2446383; doi:10.1186/1752-0509-2-55)
Supplement: Additional file 1 — SBRT Archive. An archive of the current version of the Systems Biology Research Tool. [file 1752-0509-2-55-S1.zip › sbrt-1.4.0/doc/developers_guide/api/sbrt/shell/ProcessManagerFactory.html]

ProcessManagerFactory


|  |  |  |
| --- | --- | --- |
| **Overview** \| **Package** \| **Class** \| **Use** \| **Tree** \| **Deprecated** \| **Index** \| **Help** | |  |
| **PREV CLASS**   **NEXT CLASS** | **FRAMES**    **NO FRAMES**     **All Classes** |  |
| SUMMARY: NESTED \| FIELD \| CONSTR \| METHOD | DETAIL: FIELD \| CONSTR \| METHOD |  |


---


## sbrt.shell Class ProcessManagerFactory

```
java.lang.Object
  sbrt.shell.ProcessManagerFactory
```

---

``` public class ProcessManagerFactory extends java.lang.Object ```

This class is used create `ProcessManager` objects.

**Author:**
:   This class was written and documented by
    Jeremiah Wright while in the Wagner lab.

---

| **Field Summary** | |
| --- | --- |
| `static java.lang.String` | `PROCESS`             The keyword used to denote a procedure. |
| `static java.lang.String` | `PROCESS_NAME_FILE`             The keyword used to denote a procedure name file. |


| **Method Summary** | |
| --- | --- |
| `static ProcessManager<java.lang.String,java.lang.String>` | `make(ManagerInput<java.lang.String,java.lang.String> input, java.io.PrintWriter out)`             Prints a formatted string representation of the provided input to the provided print writer and returns its corresponding process manager. |
| `static void` | `printInput(ManagerInput<java.lang.String,java.lang.String> input, java.io.PrintWriter out)`             Creates a formatted string representation of the provided input and prints it to the provided print writer. |

**Methods inherited from class java.lang.Object**

`clone, equals, finalize, getClass, hashCode, notify, notifyAll, toString, wait, wait, wait`

**Field Detail**

### PROCESS

```
public static final java.lang.String PROCESS
```

:   The keyword used to denote a procedure.

    **See Also:**: Constant Field Values

---


### PROCESS\_NAME\_FILE

```
public static final java.lang.String PROCESS_NAME_FILE
```

:   The keyword used to denote a procedure name file.

    **See Also:**: Constant Field Values


**Method Detail**

### printInput

```
public static void printInput(ManagerInput<java.lang.String,java.lang.String> input,
                              java.io.PrintWriter out)
```

:   Creates a formatted string representation of the provided input
    and prints it to the provided print writer.

    :   **Parameters:**: `input` - the input to be formatted.: `out` - the print writer to which the string will be printed.

---


### make

```
public static ProcessManager<java.lang.String,java.lang.String> make(ManagerInput<java.lang.String,java.lang.String> input,
                                                                     java.io.PrintWriter out)
```

:   Prints a formatted string representation of the provided input to the
    provided print writer and returns its corresponding process manager.

    :   **Parameters:**: `input` - a manager input containing values for `PROCESS` and `PROCESS_NAME_FILE` and all other values required to create the process manager corresponding to those two values.: `out` - the print writer to which the string will be printed. **Returns:**: the process manager corresponding to the provided input. **Throws:**: `ApplicationException` - if a process manager cannot be created from the provided manager input.


---


|  |  |  |
| --- | --- | --- |
| **Overview** \| **Package** \| **Class** \| **Use** \| **Tree** \| **Deprecated** \| **Index** \| **Help** | |  |
| **PREV CLASS**   **NEXT CLASS** | **FRAMES**    **NO FRAMES**     **All Classes** |  |
| SUMMARY: NESTED \| FIELD \| CONSTR \| METHOD | DETAIL: FIELD \| CONSTR \| METHOD |  |


---
